# Thermal Disruption of Mushroom Body Development and Odor Learning in *Drosophila*

**DOI:** 10.1371/journal.pone.0001125

**Published:** 2007-11-07

**Authors:** Xia Wang, David S. Green, Stephen P. Roberts, J. Steven de Belle

**Affiliations:** 1 School of Life Sciences, University of Nevada, Las Vegas, Nevada, United States of America; 2 Division of Biological Sciences, University of California at San Diego, La Jolla, California, United States of America; University of Maryland, United States of America

## Abstract

Environmental stress (nutritive, chemical, electromagnetic and thermal) has been shown to disrupt central nervous system (CNS) development in every model system studied to date. However, empirical linkages between stress, specific targets in the brain, and consequences for behavior have rarely been established. The present study experimentally demonstrates one such linkage by examining the effects of ecologically-relevant thermal stress on development of the *Drosophila melanogaster* mushroom body (MB), a conserved sensory integration and associative center in the insect brain. We show that a daily hyperthermic episode throughout larval and pupal development (*1*) severely disrupts MB anatomy by reducing intrinsic Kenyon cell (KC) neuron numbers but has little effect on other brain structures or general anatomy, and (*2*) greatly impairs associative odor learning in adults, despite having little effect on memory or sensory acuity. Hence, heat stress of ecologically relevant duration and intensity can impair brain development and learning potential.

## Introduction

Whereas the effects of environmental stress on developing nervous systems are well documented [1]–[3], few studies demonstrate causative influences on specific targets in the brain and their consequences for behavior. One familiar exception is the volumetric reduction of basal ganglia, cerebellum and corpus callosum due to *in utero* ethanol exposure in mammals [4]. These effects on the developing brain are associated with symptoms of fetal alcohol syndrome in humans, such as impaired verbal and visual-spatial learning, attention, reaction time, and executive functions [5]. Thermal stress is a more common and potentially hazardous feature of the natural environment for developing animals. Indeed, hyperthermia is also an especially powerful CNS teratogen in the laboratory [6], [7]. Adult male rats exposed to *in utero* hyperthermia display aberrant sexual behavior associated with disruptions of the sexually dimorphic nucleus of the preoptic area and the anteroventral periventricular nucleus [8]. However, the consequences of natural or ecologically-relevant heat stress for CNS development and function in organisms that normally experience extreme thermal heterogeneity are unknown. *Drosophila melanogaster* developing in necrotic fruit are subject to daily episodes of intense hyperthermia capable of causing significant mortality and disruption of external morphology [9], [10]. Here we show that the anatomy and function of *Drosophila* MBs, structures associated with sensory integration and higher processing in insects [11]–[13], are acutely sensitive to ecologically-relevant heat stress experienced during sub-adult stages.

Surprisingly little is known about invertebrate CNS and behavioral responses to thermal stress. In recent studies with honeybees, workers exposed to low temperatures within the range of normal experience showed reduced behavioral performance relative to their siblings raised at higher temperatures [14]. Deviations of only one degree from optimum induced striking developmental reductions in sensory mode-specific zones of the calyx, the dendritic input of the MBs [15], [16]. These findings imply that temperature-mediated MB plasticity may be important for regulating complex behavioral tasks. MBs are also remarkably responsive to sensory experience, with exposure to either enriched or deprived artificial environments inducing dramatic structural plasticity [17]–[20]. The current study expands our understanding of the acute sensitivity of the MB to stress and to thermal variation in particular. The implications of environment and experience for brain development and adult behavior are discussed.

## Results

### Heat Stress Influence on Development

**Figure 1:**
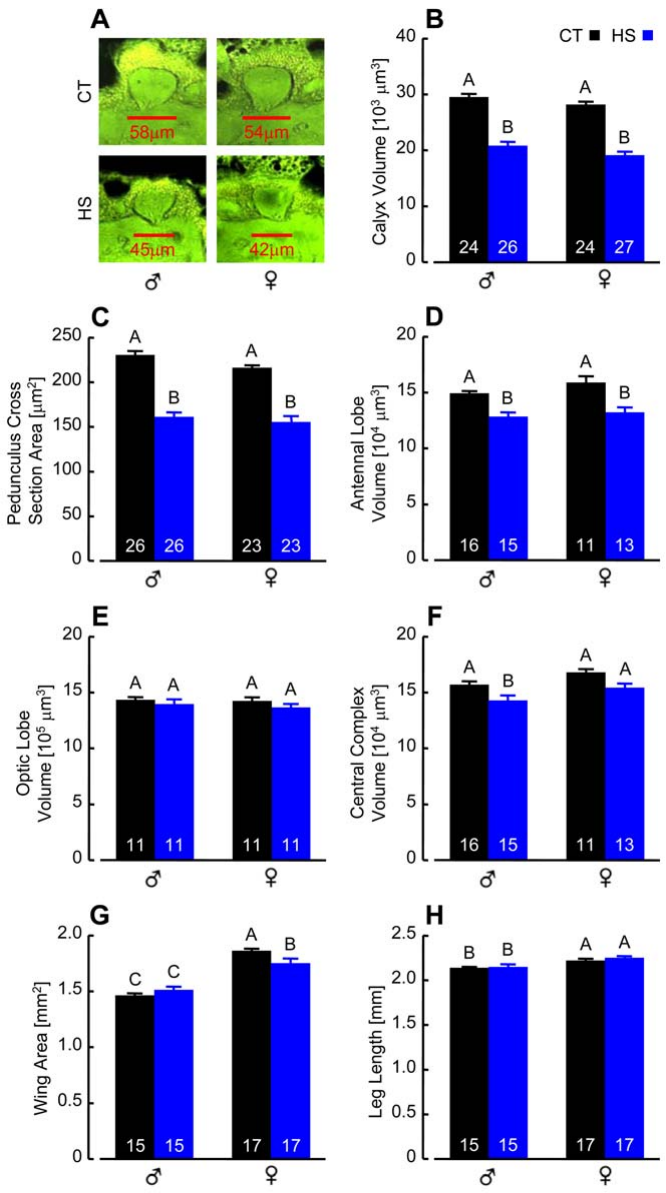
Thermal Stress Disrupts Brain Development. (A) Frontal 7 µm paraffin sections of MB calyces at their broadest point, viewed with a fluorescence photo microscope. MBs are smaller in HS flies than in the CT group. (B) Heat stress induced a significant 31% reduction in MB calyx volume (*F*_[1,97]_ = 188.39, *P*<0.0001), estimated from planimetric measurements of serial sections of HS and CT flies shown in (A). (C) MB pedunculus cross-section area (the means of measurements from three serial caudal sections) was reduced by 29% in HS flies (*F*_[1,97]_ = 123.43, *P*<0.0001). (D) AL volume [derived as in (B)] was reduced by 15% in HS flies (*F*_[1,51]_ = 26.04, *P*<0.0001). (E) Optic lobe volume [medulla+lobula, derived as in (B)] was not significantly influenced by heat stress (*F*_[1,40]_ = 1.59, *P* = 0.22). (F) Central complex volume [fan shaped body+ellipsoid body, derived as in (B)] was reduced by 9% in HS male flies only (*F*_[1,51[_ = 10.78, *P* = 0.002). (G) Wing area was reduced by 6% in HS female flies only (*F*_[1,60]_ = 7.04, *P* = 0.01). (H) Forelimb length was not significantly affected in HS flies (*F*_[1,60]_ = 1.21, *P* = 0.28). (B–H) Bars are mean±standard error (SE); *n* indicated on each bar. Different letters designate significant differences (SNK, *P*≤0.05).

*D. melanogaster* from a large orchard population reared at 23°C were exposed daily to a brief heat stress (39.5°C for 35 min) throughout larval and pupal development. This laboratory treatment mimics documented profiles of thermal oscillation experienced by developing flies in nature [9], [10], and like such intense natural hyperthermic episodes, yielded approximately 60% increases for both mortality and developmental time (data not shown). Eclosing heat-stressed (HS) adults nonetheless appeared entirely normal, with wild-type walking, flight, activity levels and reproductive capacity. However, the brains of these flies showed striking reductions in MB neuropil when viewed in paraffin sections under a fluorescence microscope (figure 1A). Using planimetric measurements to quantify this observation, we found that MB calyx volume (dendritic elements; figure 1B) and pedunculus cross section area (axonal elements; figure 1C) were both reduced by approximately 30% in HS flies relative to controls (CT) reared at a constant 23°C. In considering more peripheral brain structures associated with sensory input, antennal lobe (AL) volume was reduced by about 15% (figure 1D), while the much larger optic lobes appeared to be unaffected by heat stress treatment (figure 1E). The central complex, controlling aspects of motor output in flies and other insects [21], was 9% smaller in heat stressed males only (figure 1F). Except for a 6% wing area reduction in females, differences in external anatomical features, such as leg length, were indistinguishable between HS and CT flies (figure 1G and 1H).

**Figure 2:**
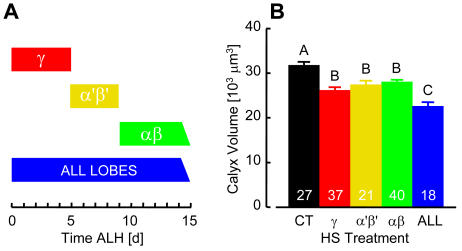
All Classes of Intrinsic MB Neurons Are Sensitive to Thermal Stress. (A) Schematic illustration of heat stress treatment administered 35 min/day throughout larval and pupal development, or restricted to specific developmental stages that correspond with the birth of MB neurons projecting to γ, α′β′, or αβ-lobes. (B) MB calyx volume measurements (derived as in figure 1B). All three classes of MB neurons are sensitive to heat stress (*F*_[4,138]_ = 17.92, *P*<0.0001). Calyx volume in flies receiving daily episodes of heat stress treatment throughout development reflected additive reductions of each of the three neuron classes exposed to heat stress as shown in (A). Bars are mean±SE; *n* indicated on each bar. Different letters designate significant differences (SNK, *P*≤0.05).

In *D. melanogaster* adults, MBs are paired neuropil structures each consisting of about 2500 intrinsic KC neurons [13], [22]. Four equivalent neuroblasts in each hemisphere of the developing brain generate three morphologically and spatially distinct classes of KCs in a specific temporal order [23]–[25]. Gamma neurons appear until the mid-3^rd^ instar larval stage, followed by α′β′ neurons until puparium formation, with αβ neurons proliferating until adult eclosion. To address whether MB hypersensitivity to heat stress might be limited to any of these classes of neurons, we examined the brains of flies that were heat stressed according to the sequential pattern of KC generation (figure 2A). Adult MBs were reduced following heat treatment during all stages of larval and pupal development, and corresponding temporal windows of KC proliferation (figure 2B). MB calyx reductions induced during γ, α′β′, and αβ neuron proliferation periods were not significantly different, suggesting that all KC classes have equivalent heat stress sensitivity.

**Figure 3:**
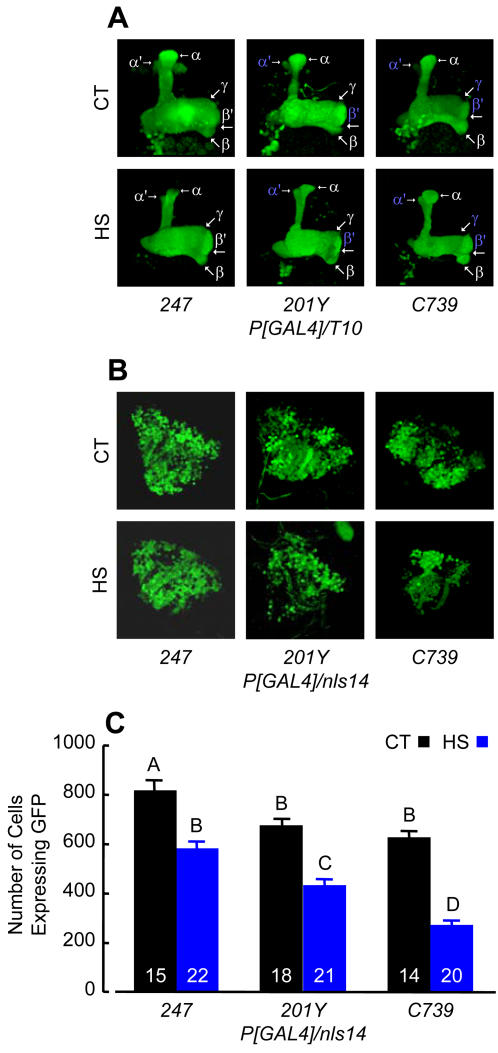
Thermal Stress Disrupts MB Development by Reducing KC Numbers. (A) Cytoplasm-targeted GFP expression patterns driven by different *GAL4*-expressing elements in whole mount brains of CT (top) and HS (bottom) flies viewed with a laser scanning confocal microscope. All MB structural elements represented in each of three CT *P[GAL4]/T10* genotypes were present (labeled) but clearly diminished in HS flies. We noted that cytoplasm-targeted GFP revealed low-level enhancer activity (labeled in blue) that is often not observed when targeting GFP expression to membranes (see references 50 and 52 for examples). (B) Nuclear-targeted GFP expression patterns driven by different *GAL4*-expressing elements in whole mount brains of CT (top) and HS (bottom) flies viewed with a laser scanning confocal microscope. We observed fewer KCs in the three HS *P[GAL4]/nls14* genotypes compared with CT flies. (C) KCs counted in the brains of flies represented in (B). A two-way ANOVA found highly significant effects of genotype (*F*_[2,104]_ = 42.36, *P*<0.0001) and treatment (*F*_[1,104]_ = 143.00, *P*<0.0001), while the interaction component was not significant (*F*_[1,104]_ = 2.69, *P* = 0.07). KC numbers were reduced by 29% in *247/nls14*, 36% in *201Y/nls14* and 57% in *c739/nls14*. Bars are mean±SE; *n* indicated on each bar. Different letters designate significant differences (SNK, *P*≤0.05).

To determine whether MB reduction in HS flies was due to either smaller or fewer KCs, we used the *GAL4/UAS* reporter gene system [26], [27] to visualize MB architecture [27]–[29] and count KC perikarya [30], [31]. In these experiments, cytoplasm-targeted green fluorescent protein (GFP) expressed by the *T10* element [32] was used to label KC projection patterns, and nuclear-localized GFP expressed by the *nls14* element [33] was used to label nuclei in KC perikarya. MBs in HS flies bearing *T10* driven by one of three different *P[GAL4]* drivers (*247* [34], *201Y* [27], or *C739* [27]) appeared slightly smaller, but otherwise normal in all respects. We observed paired neuropiles with wild-type structural features, including KC clusters, calyces, pedunculi, and lobes (figure 3A). In contrast, there were fewer labeled KCs counted in HS *P[GAL4]/nls14* flies than in CT groups (figure 3B). Cell numbers differed by 29% in *247/nls14*, 36% in *201Y/nls14*, and 57% in *c739/nls14* (figure 3C). Initially, heat stress appeared to influence numbers of GFP-expressing cells in some genetic backgrounds more than others, suggesting a possible distinction between KC classes. However, the analysis of variance (ANOVA) genotype×treatment interaction component was not significant (*F*_[1,104]_ = 2.69, *P* = 0.07), indicating that intrinsic MB neurons have similar heat stress responses. Thus, heat stress disrupts MB development by either blocking KC proliferation or triggering abnormal KC death.

### Heat Stress Influence on Behavior

**Figure 4:**
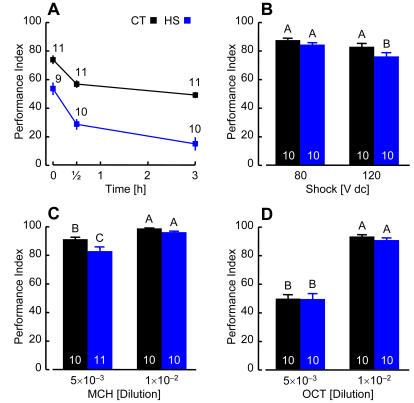
Associative Odor Learning is Impaired by Thermal Stress. (A) Olfactory learning and memory. The mean performance index calculated for HS flies was lower than CT flies at all time intervals. A two-way ANOVA detected significant effects of treatment (*F*_[1,56]_ = 101.25, *P*<0.0001) and time (*F*_[2,56]_ = 41.93, *P*<0.0001), while the interaction component was not significant; *F*_[2,56]_ = 2.00, *P* = 0.15). (B) Shock reactivity. HS flies showed normal avoidance of 80 V dc electric shock used in (A) and a slight reduction in avoidance at 120 V (*F*_[1,36]_ = 6.23, *P* = 0.017). (C) MCH odor avoidance. HS flies demonstrated a normal avoidance of MCH at the 1×10^−2^ dilution used in (A) and a slight reduction in avoidance at the 5×10^−3^ dilution (*F*_[1,37]_ = 14.72, *P* = 0.0005). (D) OCT odor avoidance. HS flies demonstrated normal avoidance responses to OCT at both dilutions. (A–D) Symbols or bars are mean±SE; *n* indicated above each symbol or on each bar. Different letters designate significant differences (SNK, *P*≤0.05).

Since MBs are a secondary olfactory neuropil essential for mediating associative odor learning and memory in *Drosophila* [11]–[13], we compared the behavior of HS and CT flies using a Pavlovian conditioning assay [35]–[37]. Learning of odors paired with electric shock was profoundly reduced (28%) in HS flies relative to CT flies (figure 4A). While memory appears to decay more rapidly in HS flies, this effect is minor since the ANOVA treatment×time interaction component was not significant (*F*_[2,56]_ = 2.00, *P* = 0.15). Performance indices averaged over all retention intervals for HS flies were 53% of the CT group. Similar olfactory conditioning defects and rates of memory decay have been described for several *Drosophila* mutants [38], [39], including those with observed reductions in MB anatomy [11], [37], [40].

Ablation studies show that *Drosophila* MBs are not required for normal responses to electric shock or noxious odors [36]. Although heat stress does have a minor influence on the development of other structures (figure 1D, 1F and 1G), and lengthens developmental time (figure 2A), HS flies did not have sensory acuity defects in control tests relevant to our conditioning paradigm. They avoided 80 V dc shock pulses normally, and responded to 120 V dc shock with only a slight reduction compared to CT flies (figure 4B). Similarly, HS flies showed normal avoidance of both 4-methylcyclohexanol (MCH) and 3-octanol (OCT) odorants at the 10×10^−3^ dilutions used in classical conditioning (figure 4C and 4D). Responses to a 5×10^−3^ dilution of MCH were slightly reduced (figure 4C). Thus, low performance of HS flies in conditioning experiments was not a secondary result of impaired shock reactivity or olfactory capacity as a consequence of AL reduction, but due to weak association of these stimuli paired during training.

## Discussion

This study demonstrates that adult *Drosophila* brain anatomy and behavior are especially sensitive to acute, ecologically relevant heat stress during development. The effect was most evident in the MBs, which were smaller due to fewer KCs, but otherwise appeared structurally normal. Calyx volume measurements in flies recently derived from a natural population and counts of GFP-labeled KCs in *P[GAL4]/nls14* brains suggested equivalent heat stress responses for all three classes of intrinsic neurons and corresponding γ, α′/β′ and α/β lobe systems. HS flies were also strongly impaired in associative odor learning, while memory decay, sensory acuity and basic motor behavior remained largely unaffected. Since odor avoidance was essentially normal in HS flies, associative functions that might be attributed to the ALs [41] were probably not markedly affected by heat stress. We saw no evidence of necrosis in paraffin sections of HS fly brains (figure 1A), and consequently favor the view that impaired KC proliferation, rather than aberrant KC mortality, was the source of MB and olfactory conditioning reduction. KCs may be especially sensitive to heat stress because they are derived from only four progenitor cells (of more than 100 in each brain hemisphere [42]) that divide asymmetrically [43] and continuously from embryo until adult eclosion [25], [44]. AL local and projection interneurons follow a similar temporal course of development [44], [45] and for this reason might be expected to show a similar sensitivity to heat stress. On the other hand, enhanced structural plasticity may be a fundamental feature of MB neurons, reflecting cellular changes that are particularly responsive to convergent sensory input, and having a profound impact on the behavioral characteristics of adults. The latter explanation may be more likely, since the optic lobes (about half of the brain) were evidently not affected by heat stress occurring throughout their development. The source of these stress response differences in the brain is a focus of our ongoing investigation.

A prevailing neural circuit model for olfactory discrimination and learning proposes that KCs serve as temporal coincidence detectors for odors paired with inherently meaningful or conditioned reinforcement [13], [46]. KCs might learn and represent odors as memories in their signaling to downstream neurons. In consideration of this model, we expect that training flies to avoid one simple odor will recruit relatively few neurons, whereas the vastly more complex natural olfactory environment should engage large overlapping KC arrays. In HS flies, fewer KCs had a diminished capacity for odor learning, but these remaining neurons had superficially normal projections and sustained relatively normal representations of odor memory. Correlated reductions of MB structure (figure 1B and 1C, figure 2B, figure 3C) and learning (figure 4A) by about 30% may reflect a simple relationship between the numbers of KCs capable of representing specific conditioned odors and learning performance, at least for the pure odorants used in our experiments. Moreover, since both MB structure and memory decay were apparently spared in HS flies, we argue that normal KC projection and connectivity are critical for memory storage and retrieval. Several observations support these simple arguments. In MB ablation studies, *Drosophila* larvae fed the cytostatic agent hydroxyurea developed into adults having only a small fraction of the normal KC complement and correlated reductions in odor learning [36]. A number of these flies had partially ablated MBs that were reduced in size but otherwise appeared anatomically normal. Similarly, mutations that reduce MB neuropil but have no obvious additional structural phenotypes also impair olfactory conditioning but not memory [37], [40]. More recent transgenic studies showed that synaptic transmission from KC terminals in the lobes is required for memory retrieval but not acquisition or storage [47], [48]. In view of these observations, we propose that lower memory scores in HS flies reflects a reduced sum of conditioned KC signals received by extrinsic neurons downstream of the MBs.

Heat stress appears to phenocopy defects described for several *Drosophila* MB anatomy mutants [11], [37], [49], providing a practical non-invasive tool for dissecting brain structure-function relationships. The significance of different KC classes, with their discrete temporal and spatial patterns of proliferation and projection to the three lobe systems of the *Drosophila* MB, is largely unknown. Mutant and transgenic studies suggest a possible distinction between them as neural substrates for representations of memories consolidated at different stages of development [18], discrete phases of memory, [28], [31], [50]–[52; see discussion in ref. 38], or conduits to extrinsic sites downstream of the MBs for memory storage and retrieval [47], [48]. Since temporal windows of heat stress can reliably induce significant and equivalent reductions of each KC class (figure 2, figure 3), this method should distinguish behavioral functions of these neurons and MB structures formed by their projections.

Although the mechanism(s) by which heat stress disrupts neural development and behavior are unknown, the apparent phenocopy of MB mutant defects may provide important clues for understanding how the brain responds to normal environmental variation. Our results suggest that KC proliferation during development is especially sensitive, while KC plasticity in adults may respond with more subtle changes [17]–[20]. Whole genome analyses (*e.g.*, DNA microarrays) should identify potential links between both types of neuronal plasticity and environmental triggers of gene activity that may either drive or accompany them.

In the wild, flies encounter stress from many sources, but also receive a broad spectrum of complementary enrichment. Stimulating environments augment MB development in a learning mechanism-dependent manner [18], while stressful environments disrupt MB anatomy and impair function. Hence, genetic influences and a combination of beneficial and deleterious environmental exposures during development likely have significant roles in determining the neural and behavioral characteristics of adults. Since all nervous systems demonstrate acute sensitivity to environmental stress, our findings have broad implications for brain development and cognitive ability in all animals, including humans.

## Materials and Methods

### Flies

Wild-type *D. melanogaster* adults were collected from a large orchard population in southern Nevada. The lineage of these flies was used for all paraffin histology and behavior. We generated heterozygous GFP-expressing flies for confocal laser scanning microscopy by crossing either *P[UAS-GFP.S65T]T10* (*T10*; Bloomington Stock Center) [32] or *P[UAS-GFP.nls]14* (*nls14*; Bloomington Stock Center) [33] with three different enhancer trap strains in which GAL4 expression was reported in distinct subsets of MB neurons: *P[Mef2-GAL4.247]* (*247*; γ, α′/β′ and α/β lobe neurons; Robert Schulz) [34], *P[GAL4]201Y* (*201Y*; γ and αβ lobe neurons; Douglas Armstrong) [27], or *P[GAL4]C739* (*C739*; αβ lobe neurons; Douglas Armstrong) [27]. Cytoplasm-targeted GFP expression was examined in HS and CT *247/T10*, *201Y/T10* and *C739/T10* heterozygotes. Nuclear-localized GFP expression in HS and CT *247/nls14*, *201Y/nls14*, and *C739/nls14* heterozygotes was used to count KC nuclei. We cultured flies at equal density in plastic vials with cotton plugs on 8 ml of standard *Drosophila* cornmeal and molasses medium at 23°C (except for heat stress treatment, below).

### Heat stress

HS treatment consisted of a single daily 39.5°C pulse for 35 min throughout larval and pupal development. We administered HS by immersing culture vials of flies in a circulating water bath. In staged HS experiments, daily heat pulses were limited to (*1*) early 1^st^ instar to early 3^rd^ instar, stressing γ-lobe neuron development, (*2*) late 3^rd^ instar to puparium formation, stressing α′β′-lobe neuron development, and (*3*) pupal development, stressing αβ-lobe neuron development, respectively.

### Histology and anatomy

We used paraffin mass histology to process flies for neuroanatomical analyses as described previously [36], [37], [53]. Three-4-day-old *Drosophila* adults were cold-anaesthetised and placed in collars. They were then fixed in Carnoy's solution, dehydrated in ethanol, embedded in paraffin, cut in 7 µm serial frontal sections, and photographed under a fluorescence microscope with an AxioCam digital camera (Zeiss). Brain structure volumes were derived from planimetric measurements of serially-sectioned brains [36], [37] using AxioVision software (Zeiss). Pedunculus cross section area was derived from the means of measurements taken from three serial sections anterior to the calyx. The means of all paired structures were used for each fly. To examine GFP expression in whole mounted fly brains, heads were dissected in PBS and maintained in Focus-Clear (Pacgen) for 15 min. They were then mounted and viewed under a fluorescence microscope with a far-blue (FITC) filter. Z-series confocal images were collected (Zeiss LSM510) to cover the whole MB for viewing structure (1.5 µm virtual sections), or perikarya clusters (0.75 µm virtual sections) for counting cells. GFP-labeled KC nuclei in HS and CT brains were counted manually in every 10^th^ section with the assistance of Image-J software [54], ensuring that all perikarya (diameters<6 µm) in each of these sections would each be counted only once.

We measured right wing area and right fore limb length to assess the effects of heat stress on external anatomy. Appendages were removed using micro scissors from cold-anaesthetised flies being processed for paraffin mass histology (above). These were mounted on glass microscope slides with cover slips sealed with nail polish. Images were photographed under a light microscope with an AxioCam digital camera and measured using AxioVision software (Zeiss).

### Behavior

Associative odor learning, memory and sensory acuity controls were assayed using a Pavlovian conditioning T-maze paradigm as described previously [35]–[37]. Groups of approximately 100 3-4-day-old flies were aspirated into a training tube embedded with an internal double-wound electrifiable copper grid. To assay odor learning and memory, flies were exposed to an air current (750 ml/min) bubbled through one odor [1×10^−2^ dilutions of either MCH (Sigma) or OCT (Sigma) in heavy mineral oil (Sigma)] paired temporally with 1.25 sec pulses of 80V dc electric shock delivered every 5 sec for 1 min. They were then exposed to an air current bubbled through a second odor without electric shock for an additional 1 min. We assessed learning and memory by presenting trained flies with both odors in converging air currents for 2 min. Performance was measured as a function of shock-paired odor avoidance at a variety of time points ranging from 1 min (giving an approximation of learning at the earliest testable time in the T-maze) to 3 hr after training. A second group of flies was trained in a reciprocal manner and tested. Scores from both tests were averaged to account for odor preferences among different populations of flies. In electric shock-avoidance controls, one arm of the T-maze was electrified with 80 or 120 V dc for 2 min. In odor-avoidance controls, flies were exposed to 5×10^−3^ or 1×10^−2^ dilutions of MCH or OCT versus air for 2 min. A performance index represents the average normalized percent avoidance of the shock-paired odor (learning, memory) or individual stimulus (sensory acuity).

### Statistical analyses

The Shapiro-Wilk test [55] showed that all 57 data samples in this report are distributed normally. Comparisons were made using ANOVA followed by the Student-Numan-Keuls (SNK) multiple range test [55] (SAS Institute software).
